# New Components of Drosophila Leg Development Identified through Genome Wide Association Studies

**DOI:** 10.1371/journal.pone.0060261

**Published:** 2013-04-01

**Authors:** Nathaniel Grubbs, Megan Leach, Xin Su, Tiffany Petrisko, Juan B. Rosario, James W. Mahaffey

**Affiliations:** 1 Department of Genetics, North Carolina State University, Raleigh, North Carolina, United States of America; 2 Department of Plant Pathology, North Carolina State University, Raleigh, North Carolina, United States of America; 3 Transgenics Department, Janelia Farm Research Campus, Howard Hughes Medical Institute, Ashburn, Virginia, United States of America; 4 Emory University, Atlanta, Georgia, United States of America; Montana State University, United States of America

## Abstract

The adult *Drosophila melanogaster* body develops from imaginal discs, groups of cells set-aside during embryogenesis and expanded in number during larval stages. Specification and development of Drosophila imaginal discs have been studied for many years as models of morphogenesis. These studies are often based on mutations with large developmental effects, mutations that are often lethal in embryos when homozygous. Such forward genetic screens can be limited by factors such as early lethality and genetic redundancy. To identify additional genes and genetic pathways involved in leg imaginal disc development, we employed a Genome Wide Association Study utilizing the natural genetic variation in leg proportionality found in the Drosophila Genetic Reference Panel fly lines. In addition to identifying genes already known to be involved in leg development, we identified several genes involved in pathways that had not previously been linked with leg development. Several of the genes appear to be involved in signaling activities, while others have no known roles at this time. Many of these uncharacterized genes are conserved in mammals, so we can now begin to place these genes into developmental contexts. Interestingly, we identified five genes which, when their function is reduced by RNAi, cause an antenna-to-leg transformation. Our results demonstrate the utility of this approach, integrating the tools of quantitative and molecular genetics to study developmental processes, and provide new insights into the pathways and networks involved in Drosophila leg development.

## Background

In the fruit fly, *Drosophila melanogaster*, development of the adult body begins when small clusters of cells are set-aside during embryogenesis to form the imaginal discs [1]–[5]. The leg primordia arise in the thoracic segments at positions where segmentally repeated expression of the signaling factor Wingless (Wg) activates expression of *Distal-less* (*Dll*) [6]. Abdominal Hox genes block *Dll* activation posterior to the thoracic segments [7], while Decapentaplegic (Dpp) and the Epidermal Growth Factor Receptor pathways limit *Dll* expression dorsally and ventrally, respectively [8].

**Figure 1:**
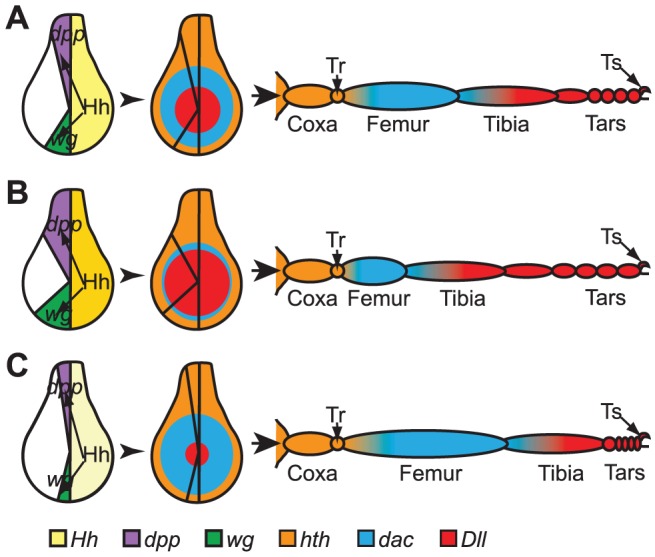
Model for larval leg disc patterning, proportionality and adult leg structure. A) Leg imaginal disc patterning begins (left) when En, present in the posterior (yellow) portion of the disc, activates *hh* expression. The Hh ligand diffuses into the anterior portion of the disc. There it activates the expression of *dpp* (purple) dorsally and *wg* (green) ventrally. Dpp and Wg, in turn, diffuse throughout the dorsal and ventral portions, respectively, of the disc, creating a gradient of their mutual presence. This gradient is responsible for the pattern of expression of transcription factors (middle) that establish the proximal-distal axis of the leg (right). In the center of the disc, mutual Dpp and Wg is highest, activating the expression of *Dll*, which is responsible for patterning the most distal structures of the leg, including the terminal structures (claw and pulvillus). Reduced mutual Dpp and Wg results in the activation of *dac*, responsible for the patterning of the middle portions of the leg. Where there is almost no mutual Dpp or Wg, Hth and Exd are active, patterning the proximal leg portions and the junction with the rest of the body. B–C illustrate how proportions might change without affecting over-all length. B) Enhanced Hh signal (dark yellow) could result in an expansion of Wg and Dpp, causing a broader domain of *Dll* expression, at the expense of *dac* (middle). This could, in turn, cause the tarsal segments to make up a larger portion of the leg without changing leg length (right). C) Similarly, the proportion of the femur could be expanded (right) if *dac* expression were expanded (middle). This might result from a decrease in expression or distribution of Dpp and Wg (left), which could, in turn, be caused by reduced Hh signal (light yellow).

After the disc primordia are established they receive patterning instructions via a series of signaling molecules, morphogens and transcription factors expressed during the larval stages that establish the proximal-distal (P-D) pattern of the legs (Fig. 1A). The *hedgehog* (*hh*) gene is activated in the posterior compartment of the disc through the action of the transcription factor Engrailed, and while Engrailed defines cells of the posterior compartment, the Hh signal is transmitted to cells of the anterior compartment where it initiates P-D development [9]. On the anterior side of the anterior-posterior border, where Hh signal is strongest, *wg* and *dpp* are activated; Dpp is responsible for dorsal fate and Wg for ventral. In addition to defining the dorsal-ventral orientation, these morphogens also work together to define the P-D axis of the leg. In the center of the disc where cells experience high levels of both Wg and Dpp, *Dll* is activated to establish the distal portions of the leg (mid-tibia through tarsus) [10]–[14]. As the mutual presence of Wg and Dpp decreases, a threshold is reached that permits the activation of *dachshund* (*dac*), which is responsible for patterning the middle leg regions (femur and tibia) [15]. Near the edge of the disc, where the mutual concentration of Wg and Dpp is lowest, *homothorax* (*hth*) is expressed. Here, the Hth protein imports Extradenticle (Exd) into the nucleus, specifying the proximal portion of the leg and its connection with the body wall [13], [16], [17]. Recently, an alternative model has been suggested proposing different molecular controls activating medial fate [4], [18], [19].

Many of the homologs of genes and genetic pathways that pattern Drosophila legs have been shown to be important in the development and growth of appendages and appendage-like structures in other metazoans [2], [3], [20]. Further, misexpression of many of these genes contributes to developmental disorders and cancer [2], [21]–[24].

Though we know many of the factors that establish or pattern appendages during Drosophila development, gaps still remain in our knowledge, gaps that, when filled, will solidify our understanding of how molecular networks establish appendage and organ development. For example, many of the genes known to be involved encode transcription factors, but their targets remain largely a mystery [2], and even some major players in leg development were missed in directed screens for appendage factors [25]. It is critical to fill these gaps to fully understand the genetic architecture of appendage development.

Like any characteristic of the adult fly, appendage morphology is a multigenic trait. Variation in expression, coding ability or mRNA stability of allelic combinations within a population will cause variation in the final morphology of the appendage, within tolerances of morphological constraints. We have taken advantage of this natural variation and employed a genome-wide association study (GWAS) using the wild-derived *Drosophila melanogaster* lines of the Drosophila Genetic Reference Panel (DGRP, [26]) to identify genes contributing to leg development. We identified single-nucleotide polymorphisms (SNPs) associated with variation in proportion of the leg segments relative to total leg length. Candidate genes were selected based on proximity to the SNPs associated with this variation and were further tested using in situ hybridization and RNA interference (RNAi) to determine whether or not the genes had a role in leg development. We identified genes from known pathways that had not been previously associated with leg development as well as previously uncharacterized genes, and demonstrated their role in this process. In addition, we identified five genes that, when expression is reduced, cause an antenna-to-leg transformation. Our results will help provide a more comprehensive view of the processes involved in appendage development in Drosophila.

## Materials and Methods

### Growth of DGRP lines

The DGRP lines were provided by the Mackay lab (NCSU, Raleigh, NC) and raised in standard environmental conditions: plastic vials with cornmeal-agar-molasses media placed in 25°C incubator. We initially screened the set of 40 DGRP lines that were to be the first sequenced. Males and females were randomly selected from each line and seven of each placed in four individual vials for egg laying. Parents were removed after three to four days depending on the amount of larval activity. We extended our screen to 100 lines (45 and 55 each) that were later sequenced to improve the power of association mapping. For these lines, 20 to 100 adults were placed in a collection cup and allowed to lay eggs on grape juice agar plates that were changed daily. After allowing an additional 24 hours for larvae to hatch, fifty randomly selected larvae were transferred to each of four fresh vials. Adult flies were preserved in ethanol 3 to 5 days after eclosing. Analysis of variance (ANOVA) indicated that there was no significant difference between flies collected in these two methods (data not shown).

### Dissection of thoracic legs and measurement

First (T1) and second (T2) thoracic legs were dissected and mounted in 70% glycerol on glass slides. Images of the legs were taken with a MicroPublisher camera (QImaging, Surrey, BC, Canada) mounted on a Zeiss AxioScope microscope (Thornwood, NY, USA) at 10× magnification. Three flies of each sex from each vial were randomly selected for leg dissection. Measurements of the legs (Fig. S1) were recorded using Adobe Photoshop and ImageJ [27]. Measurements of the femur, tibia, and tarsal segments were recorded separately (Table S1). Since the proximal end of femurs could not be seen clearly in some images, femur length was measured as the distance of the central line between the most proximal bristle socket to the distal end of the femur. Tibia length was measured as the distance between the proximal and distal ends. Each tarsal segment length was measured as the shortest distance between the ends and joint centers where the tarsus is noticeably thinner. The tarsal segments were added together to get total tarsal length, and the lengths of all segments were summed to arrive at “total” leg length.

### Statistical analysis

Factorial, mixed model ANOVAs of form Y = μ + S + L + V(L) + SxL + SxV(L) + ε were used to partition variation in leg segment proportion between sexes (S, fixed), DGRP lines (L, random), vial from which the measured fly came (V, random), the SxL interaction (random), the SxV interaction (random), and the error variance (ε) (Fig. S2 and Table S2). Association between variability in leg segment proportionality and SNP markers was tested using SAS software (Cary, NC, USA). Markers were tested for association using a simple ANOVA model: phenotype = SNP, assayed independently by position, by sex, and by leg type (i.e. T1, T2). Sites with a minor allele frequency of less than five were removed from analysis. We used data from Freeze One of the DGRP [26] for our analysis. We employed Fisher's method for combining P-values to merge data from both legs and from both sexes. We sorted by test statistics as a ranking method only; we did not convert the number to a P-value.

#### Note on our use of Fisher's method for combining P-values to sort our data

Our goal was to identify SNPs that affect general appendage development, and not those that might be sex or thoracic segment specific. Therefore, we required that SNPs affect leg proportionality in both thoracic segments and both sexes. If this were the case they would have lower P-values in all categories, both sexes and legs both thoracic segments. To sort and identify such SNPs, we calculated the summary statistic using Fisher's method of combining P-values. Fisher's method relies on independence of the traits measured, and this is not the case with our leg data. Therefore, the P-values obtained would be anti-conservative. We sorted by the summary statistic as a ranking method only, and did not utilize the P-value for any analyses. For this reason, we only report the summary statistic in our table (Table S6).

### Identifying candidate genes

From association mapping, SNP positions that had the largest test statistic were identified independently for the femur, tibia, and tarsus. During early rounds of experiments we identified a few candidates with leg imaginal disc expression (see below). We continued on with these candidates though some had somewhat lower significance as determined in later rounds of association. The GBrowse program on FlyBase version 2012_3 (www.flybase.org, [28]) was used to identify candidate genes by proximity to SNP positions. If a SNP was found between two neighboring genes, both genes were analyzed as candidates. Genes near SNPs of interest with a known function in leg development were not examined further. SNP positions greater than 8 Kbp from an annotated gene were discarded.

Potential paralogs of candidate genes were identified using BLAST [29], [30] to search the encoded protein sequence against a database of translated *Drosophila melanogaster* DNA sequences on FlyBase.

### Cloning and in situ hybridization

Genes were cloned using primers (Table S3) designed from exon sequences on FlyBase [28]. Vector NTI (Life Technologies Corporation, Grand Island, NY, USA) was used to help design primers for a coding region of each candidate gene. These regions were amplified using PCR of Drosophila genomic DNA extracted from Ore-R flies. PCR products were then cloned into a pGEM vector using the pGEM-T Easy Vector System (Promega, Madison WI, USA).

In situ hybridization was used to determine expression patterns of candidate genes in imaginal discs and embryos. Drosophila imaginal discs from third instar larvae were dissected in PBS and placed into standard in situ fixative. Digoxigenin-labeled antisense RNAs were prepared and in situ hybridization was done essentially as in [31].

### RNAi analysis

RNAi lines were obtained from the Vienna Drosophila RNAi Center (VDRC) [32] directly, and from the Transgenic RNAi Project (TRiP) from Harvard Medical School through the Bloomington Stock Center (Table S4). Males from RNAi lines were crossed to virgin females from five Gal4 lines: *rotund* (*rn*) [33], *decapentaplegic* (*dpp*) [34], *armadillo* [35], *Distal-less* (*Dll*) and *teashirt* [36]. Flies were grown at room temperature, though some were repeated at 25 degrees. Progeny from crosses were examined to determine whether or not there was an effect of silencing candidate genes in Gal4 driver domains. Efficacy of RNAi was confirmed by examining expression of the candidate gene in the RNAi larvae using in situ hybridization (data not shown).

## Results

### Quantitative variation in leg proportions of the DGRP lines

Our goal was to assess the use of the tools of quantitative genetics for identifying genes involved in Drosophila development. Variation in leg patterning is a multigenic trait, and though there will be constraints on the degree of variation, if measureable, we could use statistical analyses to map candidate loci contributing to that variation. Though previous forward genetic studies have revealed a wealth of information on appendage development in Drosophila, the use of natural variation offered a unique perspective with different sensitivities. We chose to examine variation in proportion of leg segments to bring into focus current models of leg patterning (Fig. 1). For example, a small change in the strength of Hh, Dpp or Wg signals could alter the expression of determinant genes (*hth*, *dac*, or *Dll*), which, in turn, could shift the boundaries of leg segmentation and, hence, change proportioning without necessarily affecting over-all length. Therefore, variation in proportion of the segments (femur, tibia, tarsus) seemed the more informative trait to study if we wanted to identify genes that might affect early appendage patterning. Further, this would remove or reduce other influences, such as variation in metabolism or growth rate.

We dissected legs from first (T1) and second (T2) thoracic segments measuring twelve individuals of both sexes from 137 DGRP lines [26], [37]. Of these, 117 were included in the Freeze 1 sequences of the DGRP (Fig. S1). The proportion of each segment was calculated as compared to the total as defined in Materials and Methods. Statistical analyses indicated strong correlations between leg segments of both thoracic segments and both sexes within a line, as we would expect if the variation were due to alleles of general appendage-patterning genes (Table S5). Further, there were significant correlations between most of the segments within a leg (femur, tibia and tarsus, Table S5), though the correlation between proportion of tibia and of femur was relatively low (not significant in male T1). In addition, the correlation between the tarsus and other segments was negative, which might be expected if expanding the proportion of one segment would require a simultaneous reduction in another. The variation in proportionality for the leg segments for males and females of the 117 DGRP lines is shown in Fig. S2.

### GWAS and candidate gene selection

The top 20 SNP positions associated with variation in each of the three leg segments, along with the genes identified as closest to the SNP are listed in Table S6. (FlyBase numbers for most candidate genes are included in the Table S6. Those not in the table are included in the text.) Although some SNPs were not located near any known or predicted genes, many SNP positions were found in introns, exons, and protein coding regions, as well as just upstream or downstream of genes. Several of the genes neighboring or harboring the SNPs were already known to be involved in leg development, including *Moesin* (*Moe*) [38], *Enhancer of zeste* (*E(z)*) [39], [40] and *myospheroid* (*mys*; FBgn0004657) [41]–[43] or suspected to be, *disco-related* (*disco-r*) [25]. Identification of these genes helped to validate our methods. Some candidates were named genes with known functions, but their role in leg development had not been examined. These were selected for further study to assess whether they also had roles in leg development. Some candidate genes had not been previously characterized.

### Expression of candidates during Drosophila development

**Figure 2:**
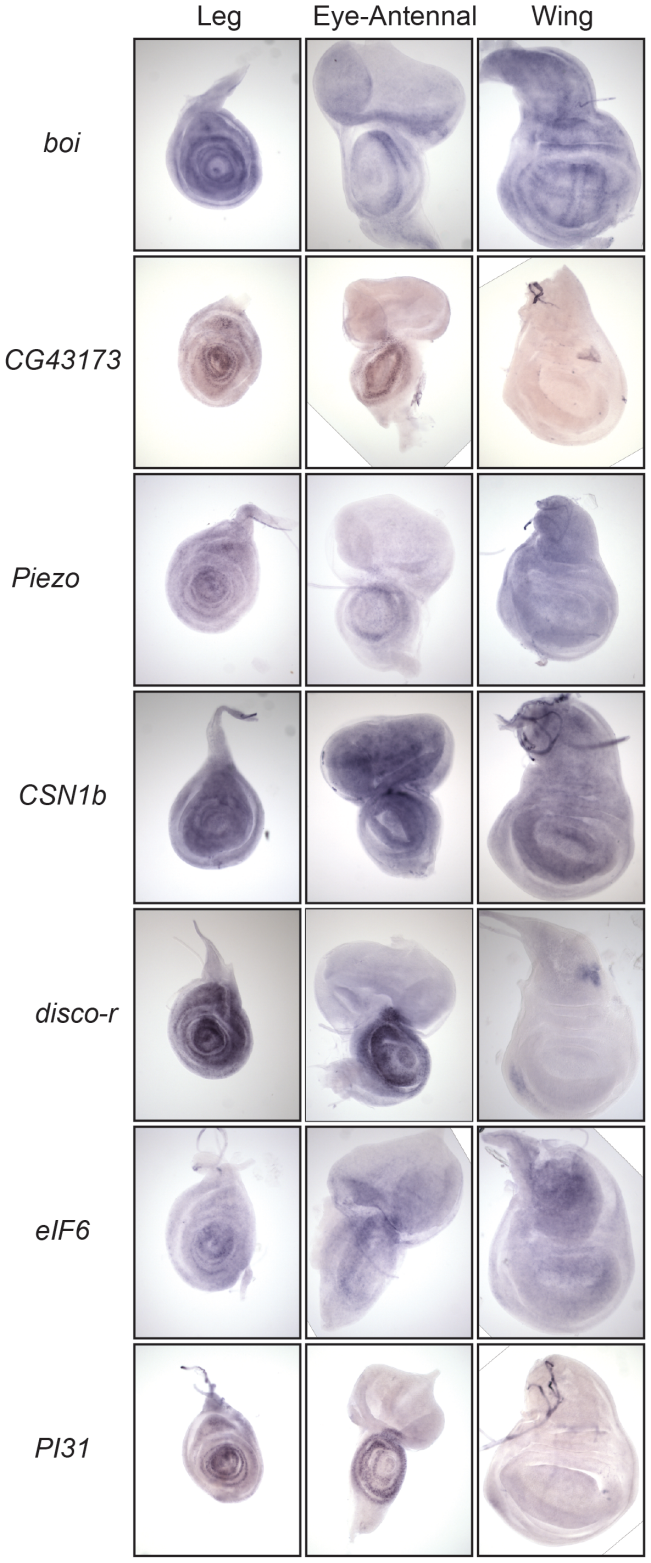
Distribution of candidate gene mRNA in imaginal discs. Wild-type third instar imaginal discs stained to detect mRNAs from several of our candidate genes. Examples with several different mRNA distribution patterns are shown. There are several that are of note, the cross-like pattern of *boi* in the wing disc that borders *wg* and *dpp* expression, the narrow tarsal staining of *eIF6*. We also note that *disco-r* is expressed in the ventral edge of the wing which had not been reported previously.

To determine if our candidate genes played a role in leg development, we first examined distribution of mRNAs encoded by these genes using in situ hybridization, looking for expression in the leg imaginal discs of larvae or their primordia in embryos (Fig. 2 and Table S7). Of the 37 genes examined, we did not detect staining in the leg imaginal discs for seven (*CG5549*, *M-spondin* (*mspo*), *dpr8*, *CG7949*, *CR33218*, *CG42353*, *CG42354*). Of these, only *mspo* and *CG7949* had embryonic expression, but neither was expressed in the disc primordia. These seven genes were excluded from further analysis.

Transcripts from several genes accumulated in broad regions of the leg discs, such as *brother of ihog* (*boi*) and *eIF6*, while others were enhanced in the presumptive tarsal region, like *CG43173* and *PI31* (FBgn0033669). We also observed staining for many of these genes in the developing antennae, where some were also patterned. This is not unexpected as the antennae and legs are serially homologous structures [44]–[48]. Transcripts for some genes were observed in the wing, haltere and eye as well. Of particular note was the Hedgehog pathway member *boi*, which, in the wing discs, had enhanced staining in the wing blade region outlining the expression domains of *wg* and *dpp* (Fig. 2). We observed staining in embryos with 17 of the 30 probes that had expression in the imaginal discs; however, with the exception of *disco-r* [49], none of these appeared to have staining specifically in the developing disc primordia (data not shown).

### RNAi phenocopies in appendages

To determine whether any of our candidate genes were necessary for normal leg development, we used RNAi to reduce gene function of those candidates with mRNAs detected in the leg discs. We used transgenic RNA interference lines from the Vienna Drosophila RNAi Center (VDRC) [32] and Transgenic RNAi Project (TRiP) with several Gal4 drivers (see Materials and Methods). However, the *Dll*-Gal4 driver [36], expressing Gal4 in the medial-to-distal portion of the leg discs, was most informative, so results described below (and summarized in Table 1) are primarily from the use of this driver (referred to as *Dll*-RNAi below). RNAi lines were not available for four candidate genes (*yantar*, *GG43173*, *CG43174* and *Hexosaminidase 1* [FBgn0041630]).

**Table 1:** Summary of phenocopy from RNAi of listed gene driven by listed Gal4 driver.

Gene	armGAL4	DllGAL4	Associated segment
*bnl*	Lethal	No effect	Tibia
*boi*	No effect	No effect	Femur
*CG10947*	No effect	No effect	Femur
*CG13707*	No effect	No effect	Tarsus
*CG15012*	No effect	No effect	Tarsus
*CG18317*	No effect	No effect	Femur
*CG30371*	No effect	Antenna-to-leg	Tibia
*CG32333*	No effect	Antenna-to-leg	Tibia
*CG3961*	No effect	No effect	Femur
*CG43444*	No effect	No effect	Femur
*CG6841*	No effect	Pupal lethal; tarsus only have a few, fused segments; antennae reduced	Tarsus
*CG7695*	No effect	No effect	Tibia
*CG9129*	No effect	Antenna-to-leg	Tibia
*CG9134*	No effect	No effect	Tibia
*chinmo*	Lethal	Lethal	Tarsus
*Cka*	Lethal	Tarsus deleted, arista and antenna segment 3 deleted, wings hooded	Tarsus
*ckn*	No effect	No effect	Tarsus
*CSN1b*	No effect	Legs and sex combs reduced, arista reduced, wings hooded	Tarsus
*disco-r*	No effect	Antenna-to-leg	Tarsus
*eIF6*	No effect	Pupal semi-lethal; tarsi deleted and sex combs reduced	Femur
*GlcT-1*	No effect	No effect	Femur
*mys*	Lethal	Lethal	Tarsus
*PI31*	No effect	Legs missing one tarsal segment, arista deleted/reduced, wings hooded	Femur
*Piezo*	No effect	Antenna-to-leg	Tibia
*RYBP*	No effect	No effect	Tarsus
*sick*	No effect	Lethal	Femur

FlyBase IDs: *CG10947*, FBgn0032857; *CG15012*, FBgn0035528; *GlcT-1*, FBgn0067102.

**Figure 3:**
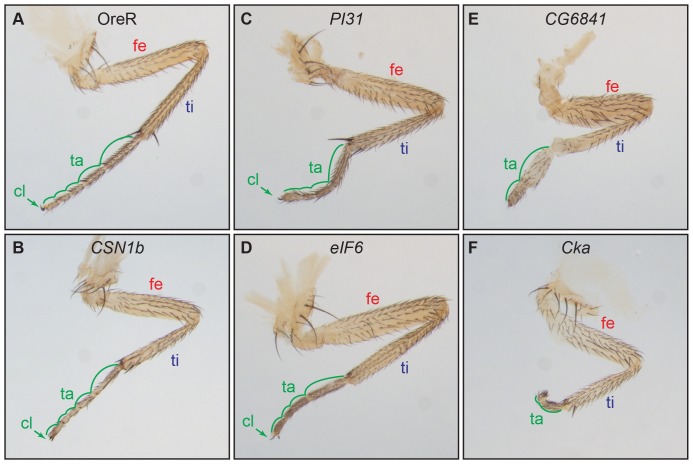
*Dll*-Gal4 driving RNAi phenocopies in legs. Second thoracic legs from female flies with *Dll*-Gal4 driving UAS-RNAi insertions. All flies were grown at room temperature. A) Wild-type. B) RNAi of *CSN1b* resulted in reduced leg size, but did not alter shape. C) RNAi of *PI31* reduced length of tarsal segments, with occasional loss of a single distal segment. D) RNAi of *eIF6* caused deletions and fusions of tarsal segments. E) RNAi of *CG6841* deleted most of the tarsal segments and terminal structures. F) RNAi of *Cka* was similar to that of *CG6841*, but more severe. Abbreviations: fe, femur; ti, tibia; ta, tarsal segments; cl, claw.

**Figure 4:**
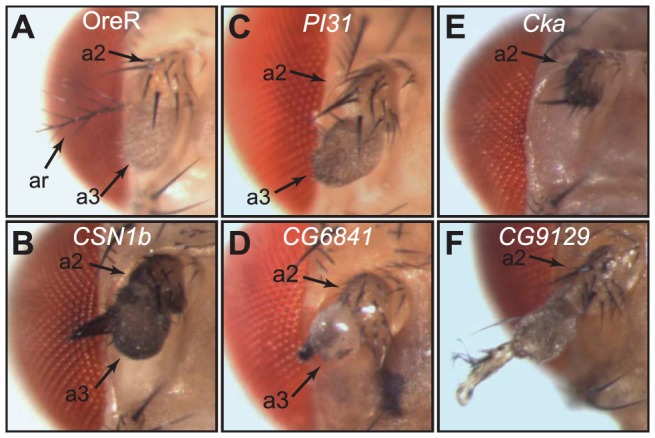
Effect in antennae of *Dll*-Gal4 driving RNAi. Heads from flies with both *Dll*-Gal4 and UAS-RNAi insertions that disrupted antennal development. Arrows with numbers indicate the antennal segments. A) Wild-type. B) RNAi of *CSN1b* resulted in loss of arista, and appearance of sclerotized structure C). RNAi of *PI31* resulted in loss of arista while the remaining structures appear normal. D) RNAi of *CG6841* resulted in loss of arista, and drastic changes to the appearance of antennal segment 3, and some reduction of A2. E) RNAi of *Cka* resulted in loss of all but proximal structures. F) RNAi of *CG9129* is characteristic of the five lines that resulted in transformation of antennae toward leg identity beginning in distal A2, though slight differences were noted between the five lines that caused these transformations.

We observed altered leg morphology with six candidate genes. At room temperature, *Dll*-RNAi with *COP9 complex homolog subunit 1b* (*CSN1b*) resulted in smaller legs relative to overall body size (Fig. 3B) and occasional loss of the fifth tarsal segment. These flies also had reduced aristae in the antennae (Fig. 4B), and males had reduced sex-combs (data not shown). At 25 degrees reduction of *CSN1b* caused loss of multiple tarsal segments. *Dll*-RNAi of *PI31* reduced the relative size of tarsal segments, occasionally deleting one of the middle tarsal segments (Fig. 3C), and also deleted the aristae in the antennae (Fig. 4C).

*Dll*-RNAi of *eIF6*, *CG6841* and *Connector of kinase to AP-1* (*Cka*) each resulted in fused and deleted tarsi, with the later two also deleting terminal structures, the claws and pulvilli (Fig. 3D–F). In addition, *Dll*-RNAi of *eIF6* caused reductions in the sex-comb tooth number (data not shown). RNAi of *CG6841* and *Cka* also altered or deleted distal antennal segments (Fig. 4D, E).

In some cases *Dll*-RNAi was lethal. For example, *Dll*-RNAi with *sickie* (*sick*) was lethal prior to pupal formation, so we could not determine if there was an effect on leg development with that driver. However, with the *rn* driver (*rn-*RNAi, expressing Gal4 in the distal portion of the leg and in the wing blade region) reducing *sick* had no apparent effect in the legs, except with sex-comb tooth numbers in males, which were either greatly decreased or increased (data not shown). Reduced expression of *bnl* (FBgn0014135) and *Chronologically inappropriate morphogenesis* (*chinmo*) caused early death with multiple drivers.

RNAi of many candidates, driven by either *rn*- or *Dll*-Gal4, also resulted in altered wing morphology, ranging from a hooded phenocopy with slight reduction in size accompanied by a downward curvature, to near total loss of wing blade (data not shown).

Somewhat surprisingly, *Dll-*RNAi with five candidates (*CG9129*, *CG32333*, *CG30371*, *Piezo* [FBgn0031993] and *disco-r*) caused antenna-to-leg transformations (for example see *CG9129* in Fig. 4F), yet no overt disruption of leg morphology was observed with these candidates. Since all five RNAi lines were from the VDRC we were suspicious that this could be due to a background effect with some of the VDRC RNAi lines. To test this, we generated flies with the RNAi insertions and *Dll-*Gal4, with and without *tubulin-*Gal80 on the X chromosome. Presence of Gal80 would block activation of the RNAi construct, so if the antenna-to-leg transformations were due to a genetic background effect in some of the VDRC lines, then we would expect to observe the transformation regardless of whether Gal80 was present. However, presence of Gal80 completely blocked the antenna-to-leg transformation indicating that the transformations were due to activation of the RNAi constructs, likely through reduction of the candidates.

## Discussion

### General considerations of the GWAS approach for identifying genes important for limb development

Studies of appendage development in Drosophila have been extremely valuable in understanding the genetic networks governing many aspects of organ and structure development. Further, comparative studies in other organisms have opened the door for examining how genetic changes lead to diversification of structures and, thereby, evolution. GWAS takes advantage of the natural variation in a trait or phenotype to identify genes that contribute to the development of that trait. It is most important to understand that it is the combination of many genes with small effect that gives rise to the natural variation of a trait. Hence, a large effect is not expected from any one SNP. By associating a particular SNP with variation in the trait of interest, we can map the positions of genes that contribute to the trait. Thus GWAS offers a different perspective on gene identification and can circumvent issues affecting more standard forward genetic screens, such as early lethality and redundancy. Although GWAS has been used in the past to locate genetic causes of variation in behavioral and quantitative traits and causes of heritable diseases [50], [51], it has not, to our knowledge, been employed to identify genes involved in developmental processes. Our study demonstrates that integrating the tools of Quantitative and Developmental Genetics can be very productive, addressing issues of complex genetic interactions governing development.

The presence of functionally redundant paralogs in the genome could mask a leg development role in most forward genetic analyses. From our GWAS, we identified several candidates where a paralog is known or suspected (Table S8). Of these, *boi*, in particular, stands out. *boi*, and its paralog, *interference hedgehog* (*ihog*, FBgn0031872) have recently been shown to encode proteins critical for Hedgehog (Hh) signaling during Drosophila wing development [52]–[59]. Currently, neither has been implicated in leg development. However, since Hh signaling is critical in the legs, it is reasonable that these genes could play a part in leg development and that SNPs in these genes could contribute to quantitative variation in leg phenotypes. Therefore, it is not surprising they would show up in our GWA Study. Boi and Ihog bind Hh, strengthening the activation of the Hh signaling pathway, while limiting the range of the ligand [55], [56]. In the leg discs, any change that broadens or narrows the range of Hh could, in turn, broaden or narrow the expression domains of the downstream responders (*wg* or *dpp*), thereby altering the domains of expression of the transcription factors responding to these signals (Fig. 1). While this potential connection to the leg development model makes *boi* an intriguing candidate to pursue, reduction of *boi* (or *ihog*) alone (through *Dll*-RNAi) caused no obvious malformation of legs or antennae. We suspect that the redundancy of these genes prevented any gross abnormalities in leg development. Presumably, subtle tweaks to *boi* expression or function could have effects on leg patterning within the realm of a multigenic characteristic of a population.

Redundancy might also have an impact on the antenna-to-leg phenocopy we observed with five candidate genes (*CG9129*, *CG32333*, *CG30371*, *Piezo* and *disco-r*, see Table S8). We suspect that redundancy is the reason that we did not observe malformation of the legs simultaneously with the antenna-to-leg transformations, and that these candidates actually do have roles in leg development. This is certainly true for *disco-r*, where the known paralog, *disconnected* (*disco*) masks the role of loss of *disco-r* alone during leg development [25], [49]. Perhaps, antennae are more sensitive to RNAi of one particular paralog versus the other, or that the antennae are more sensitive than the legs to the reduced *Dll* function of the *Dll*-Gal4 mutation in combination with the RNAi (see below). Indeed, we have noted that the RNAi process appears to be more robust in the antennae than in the legs (data not shown). Because of redundancy, it is possible that neither *boi* nor the antenna-to-leg candidates would easily have been discovered from standard forward genetic screens. This demonstrates the power of GWAS as a tool to overcome some of the limitations of more traditional methods of identifying genes involved in developmental pathways.

As a final comment about target gene identification from our GWAS study, we do expect some false positives in our analysis. An estimated False Discovery Rate (FDR) indicates that approximately 20 SNPs could have arisen by chance using a cutoff of 1e-5 (Table S9). However, because this FDR estimation assumes all tests are independent, it is an overestimate. Lack of independence arises because of linkage disequilibrium between neighboring SNPs, and this would mean that the actual number of analyzed SNPs is somewhat lower than that used for the FDR estimation. Hence, the false discovery estimate is inflated.

### New insights into the pathways of appendage development

In Table 2 we summarize what is known about the proteins and their functions of our candidates. Though a few of these encode known proteins, many are computed genes (CGs) at this point, so the data obtained from this study will help in defining pathways associated with these genes.

**Table 2:** Summary of protein functions.

Gene	Protein Domains and Functions	Segments
*bnl*	Tracheal system development; Cytokine and Heparin-binding growth factor/Fibroblast growth factor domains.	Tibia
*boi*	Hedgehog pathway member with Fibronectin and Immunoglobulin domains.	Femur
CG30371*	Possibly involved in proteolysis, and contains multiple peptidase domains.	Tibia
CG32333*	Two domains of unknown function, DUF3657 and lipase-like DUF676.	Tibia
*CG43444*	Contains a CXXC-type zinc finger.	Femur
*CG6841*	Involved in regulation of alternative nuclear mRNA splicing, via spliceosome and in neurogenesis.	Tarsus
CG9129*	Unknown molecular function and processes.	Tibia
*chinmo*	Contains a C2H2-type zinc finger domain and BTB/POZ domain.	Tarsus
*Cka*	Involved in dorsal closure and positive regulation of JNK cascade. Contains G-protein beta WD-40 repeat.	Tarsus
*CSN1b*	Contains a 26S proteasome regulatory subunit.	Tarsus
disco-r*	Contains a C2H2-type zinc finger domain.	Tarsus
*E(z)*	Histone methyltransferase activity, involved in a number of developmental processes, including leg development.	Tarsus
*eIF6*	Translation initiation factor activity.	Femur
*Moe*	Protein binding protein involved in a number of developmental processes, including leg development.	Femur
*mys*	Epidermal growth factor protein.	Tarsus
*PI31*	Positive regulation of proteasomal ubiquitin-dependent protein catabolic process.	Femur
Piezo*	Mechanically-gated ion channel activity.	Tibia
*sick*	Involved in defense response to Gram-negative bacteria.	Femur

Known and predicted functions of proteins were obtained from Flybase and are provided only for genes with phenocopy (*boi* was included because of its known redundancy with *ihog*). The five candidates that gave antenna-to-leg transformation with the *Dll* driver are marked with an asterisk.

Three candidates potentially could be involved in expanding or restricting the signals or morphogens involved in leg development (*boi*, *CSN1b*, *mys*). From the model shown in Fig. 1, it is easy to understand how altering components of Hh signaling could contribute to changes in leg proportionality. As described above, *boi* is known to function in the Hh pathway. *CSN1b* encodes a subunit of the COP9 signalosome/ubiquitin-proteasome system, which has recently been implicated in Hh signaling in the wing [60]. However, the role of this highly conserved signalosome [61], [62] could be broader, as a potential function in transcription regulation [63]. The Drosophila *mys* gene encodes a homolog of vertebrate beta PS integrin [42], [43], [64], which is involved in cell adhesion and signaling. Loss of *mys* leads to loss of portions or all of the leg [41].

PI31 might also be involved in signal modulation. PI31 is a highly conserved regulator of proteasome function [65], [66]. It has both inhibitory and activation properties, depending upon the test system. In Drosophila, PI31 binds to the F-box factor, Nutcracker to activate proteasome and caspase during sperm maturation. It is also active in cell cycle regulation. Perhaps, like CSN1b, PI31 might be involved in a signal modulation that involves the proteasome.

Several genes identified in our GWAS encode proteins with transcription factor domains (Table 2); particularly prevalent were genes encoding Zn-finger transcription factors (*CG43444*, *disco-r*, *chinmo*). At present, we do not know whether these factors might function upstream of the Hh signal or are integrated into the response. We do know that ectopic expression of *disco-r* or its paralog *disco* can induce ectopic ventral appendage structures [25], and that cells require at least one of these genes to form ventral appendages (J.R and J.W.M. unpublished). Further work will be required to discern where these other genes fit into the appendage network.

The classic antenna-to-leg transformation arises from ectopic expression of the homeotic gene *Antennapedia* [45], [67]. Hth is central to antennal specification and, therefore, the transformation. When Hth and Exd are present in the same cells, Hth helps recruit Exd to the nucleus, inducing antennal fate. If Hth is reduced, Exd remains cytoplasmic and the antennae develop leg-like characteristics.

Only a few genes are known where recessive mutations cause this transformation, *Dll*, *hth* [11], [68], *exd* [69] and *spineless* [70]. Ectopic Antennapedia or reduction of either Dll or Spineless reduces expression of *hth*, thereby decreasing import of Exd into the nucleus. Given this rather straightforward model for antennal specification, we were quite surprised to find that five different RNAi lines caused an antenna-to-leg transformation when driven with Dll-Gal4. However, since ectopic expression of *disco-r* can induce ectopic antennae in the eyes (J. W. Mahaffey, unpublished), it is perhaps not surprising that reduction of *disco-r* can cause this transformation.

The only other gene of our antenna-to-leg candidates with a postulated function is *Piezo*, which encodes a mechanically gated ion channel thought to be involved in reception of mechanical stimuli [71], [72]. How this and the other uncharacterized genes integrate into antennal, and possibly leg, development remains unknown. Perhaps it is worth noting that recent data indicate that Piezo is involved in tissue homeostasis, functioning to extrude cells from epithelia under crowded conditions [73].

An important point to consider with the antenna-to-leg transformations is that the Gal4 insertion of *Dll*-Gal4 creates a hypomorphic allele of *Dll*, *Dll^md13^* [36]. While *Dll*-Gal4 alone does not cause an antenna-to-leg transformation, this mutation could generate a sensitized background for our candidate RNAi, so it is possible that the transformations we observe are the result of a synergistic effect between reduction of the candidate and reduced Dll. Though we do not know what, if any, contribution *Dll*-Gal4 has on the transformation, inclusion of Gal80 in our experiments demonstrates that the RNAi activation was required, suggesting that these genes are likely involved in the “antenna or leg” genetic decision.

**Figure 5:**
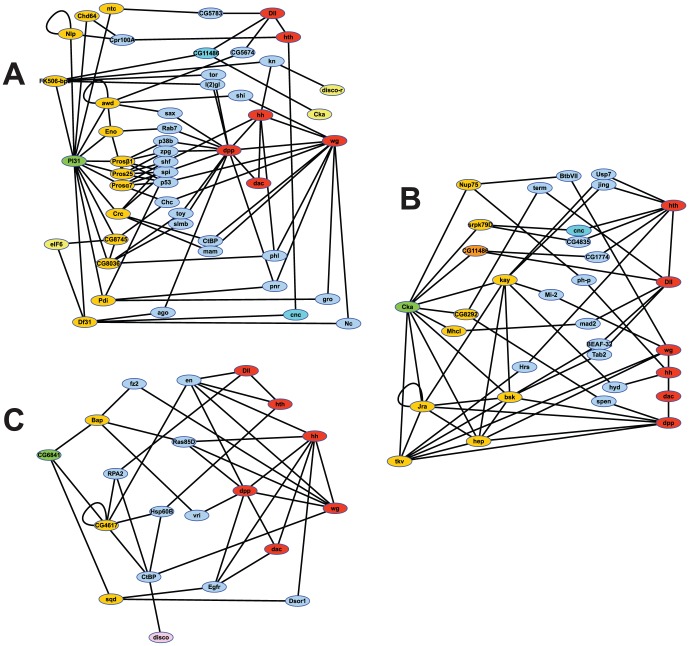
DroID analyses identifying connections between candidates and canonical leg development genes. Genes in orange connect directly to the candidate (Green), and may be linked directly or through genes in blue to the canonical appendage cascade genes (red). Yellow identifies those of our candidate genes that are linked to the test gene. A) Analysis of PI31. Links shared with Cka are shown in dark blue. B) Analysis of Cka. Links shared with PI31 are shown in dark orange and dark blue. C) Analysis of CG6841, including a link to disco in pink.

The genes we have identified in this study might be expected to participate in some way with other genes known in the appendage developmental network (for example, *hh*, *wg*, *dpp*, *Dll*, *dac*, and *hth*), and we thought perhaps that we could identify possible network links using programs such as DroID [74]. These programs provide a mechanism to search for potential interactions discerned from prior genetic research and genome wide studies of protein interactions. We used DroID and Cytoscape [75] to search for known and predicted protein-protein or genetic interactions that could link our candidates to the canonical appendage development network. There was little information for many of our candidates, which was not surprising since they are CG's; however, three networks predicted for *Cka*, *PI31* and *CG6841* were informative and are shown in (Fig. 5). These three genes were two steps or less from the canonical genes mentioned above, though *dac* was an exception sharing few, if any, connections. Not surprisingly, a network of proteasome-related genes connected *PI31* to the canonical pathway (Fig. 5A); this may support our hypothesis that PI31 could play a role in regulating signaling processes via protein degradation. *PI31* also shared connections with other of our candidates, *eIF6*, *Cka* and *disco-r*. *Cka* (Fig. 5B), the RNAi of which caused a strong phenocopy in the leg, was linked to the canonical leg pathway through a number of genes encoding kinases, including members of the JNK pathway. The role of Cka in the JNK pathway has previously been shown to be important in the proper expression of *dpp* during dorsal closure [76], so similar mechanisms may play an important role in Dpp function during leg development. Another candidate with a strong RNAi phenocopy, *CG6841* (Fig. 5C), has not been extensively studied, yet this network analysis identified several connections to the pathways of known leg development genes, including *disco* and *engrailed*, an early component of the cascade [9]. These links support the usefulness of our method, and perhaps can be used to identify other novel leg development genes.

We have shown that Genome Wide Association Studies provide a useful method for identifying genes contributing to a developmental process. Further, we have evidence that this method can overcome some of the limitations of traditional developmental genetic screens, such as redundancy. We demonstrate this by successfully utilizing a GWAS screen to identify several new components of signaling, transcription and unknown pathways that contribute to Drosophila appendage development. Thus, we believe these results show the applicability of this method to any developmental trait. All that is required is measurable variation in morphology of a relevant trait arising from the developmental process targeted. This method should prove beneficial for extending what is known about even well studied developmental genetic pathways.

## Supporting Information

Figure S1 **Measuring legs.** An example DGRP female, T2 leg marked to show the measuring method. In the femur, a black circle marks each of the two most proximal bristles. Measurement begins in the middle of the line (black) between these two bristles and ends at the end of the segment (red line). The tibia is measured from its beginning, in the joint with the femur, and ends where it forms a joint with the first tarsal segment (blue line). Each tarsal segment was measured separately (green lines), beginning with the joint to the more proximal segment and ending with the joint to the more distal segment (green arrow heads). In the case of the first tarsal segment, measurement began at the joint with the tibia. Measuring the final tarsal segment ended at the tip of the leg, not including terminal structures.
(TIF)

Figure S2 **Variation in proportions of leg segments.** Mean leg segment proportion for each measured DGRP line is graphed on the Y-axis, organized from smallest to largest based on female values (red) for each trait. Males are shown in blue. The P-value for line effect is also shown, and was very significant in all cases.
(TIF)

Table S1 **Leg measurements.**
(CSV)

Table S2 **ANOVA of leg proportions.**
(XLS)

Table S3 **Primers for gene cloning.**
(XLS)

Table S4 **Source of RNAi Lines.**
(XLS)

Table S5 **Correlations between traits.** Degree of significance from the lowest possible value (0) and the highest possible value (1) were also calculated (recorded in columns ‘t’ and ‘P-value’). Fe, femur proportion; Ta, tarsus proportion; Ti, tibia proportion; F, female, M, Male; A, both sexes; T1, first thoracic leg; T2, second thoracic leg.
(XLS)

Table S6 **Top candidate genes.** Candidates were sorted using Fisher's method for combining P-values to identify SNPs with the highest summary statistic (Fishers) in all four measurements (T1 and T2 and Males and Females). Candidate genes were identified by proximity to SNPs In cases where multiple SNPs identified a candidate, only the most significant SNP position is shown. FlyBase IDs are included in this table.
(XLS)

Table S7 **Observed expression of analyzed candidates.** * = selected early, † = previously published, P = patterned expression, U = ubiquitous expression.
(XLS)

Table S8 **Possibility of paralogs.** Probable = Genes with potential paralogs. Genes with known redundant paralogs are labeled as “Yes” along with the name of the paralog. The five candidates that gave antenna-to-leg transformation with the Dll driver are marked with an asterisk.
(XLS)

Table S9 **False Discovery Rate Estimate.**
(XLS)
