# Supplementary figures and images for: New Components of Drosophila Leg Development Identified through Genome Wide Association Studies
Source: PLoS One. 2013 Apr 1;8(4):e60261. doi: 10.1371/journal.pone.0060261 (PMC3613359; doi:10.1371/journal.pone.0060261)

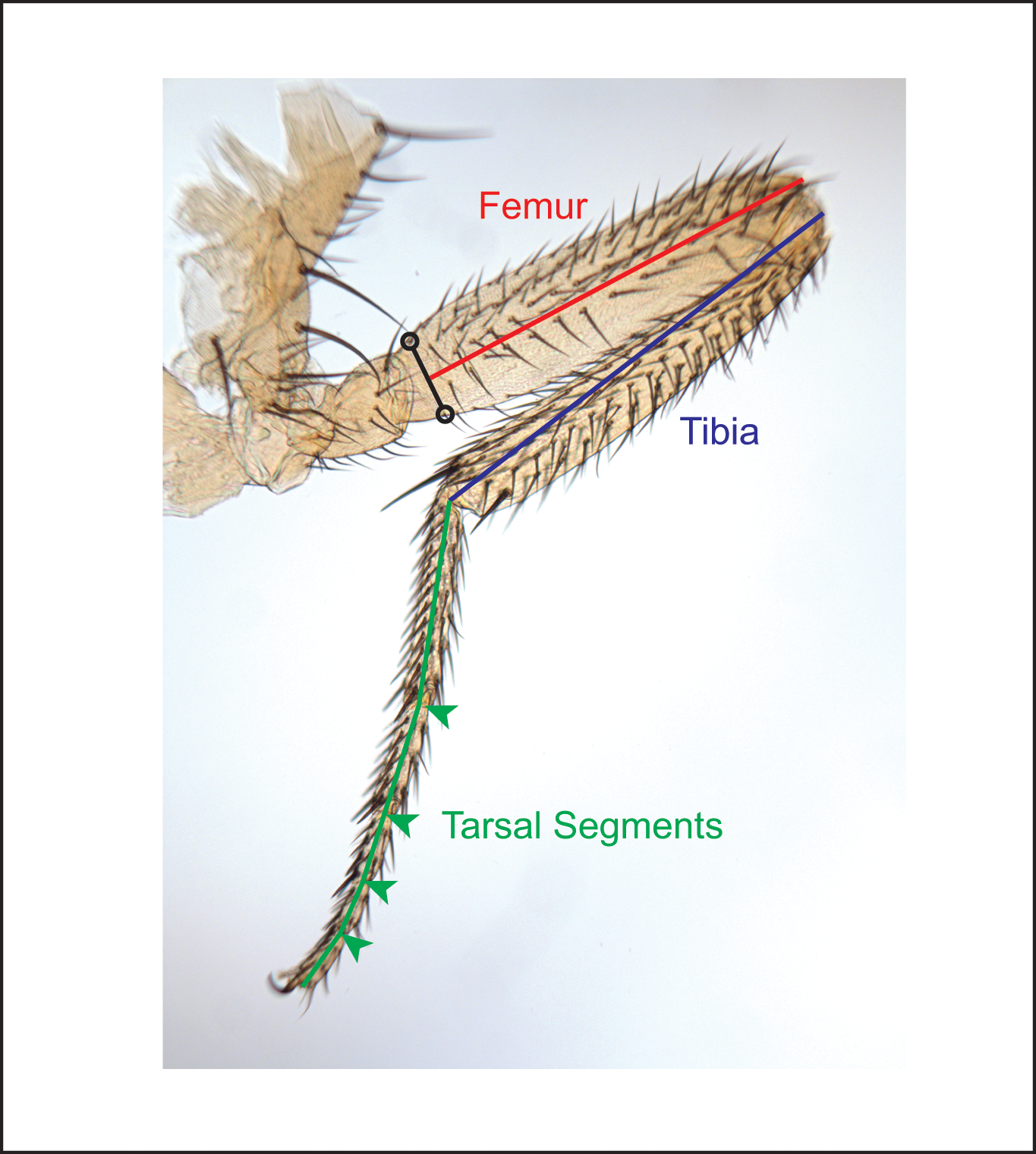

Supplement: Figure S1 — Measuring legs. An example DGRP female, T2 leg marked to show the measuring method. In the femur, a black circle marks each of the two most proximal bristles. Measurement begins in the middle of the line (black) between these two bristles and ends at the end of the segment (red line). The tibia is measured from its beginning, in the joint with the femur, and ends where it forms a joint with the first tarsal segment (blue line). Each tarsal segment was measured separately (green lines), beginning with the joint to the more proximal segment and ending with the joint to the more distal segment (green arrow heads). In the case of the first tarsal segment, measurement began at the joint with the tibia. Measuring the final tarsal segment ended at the tip of the leg, not including terminal structures. (TIF) [file pone.0060261.s001.tif]

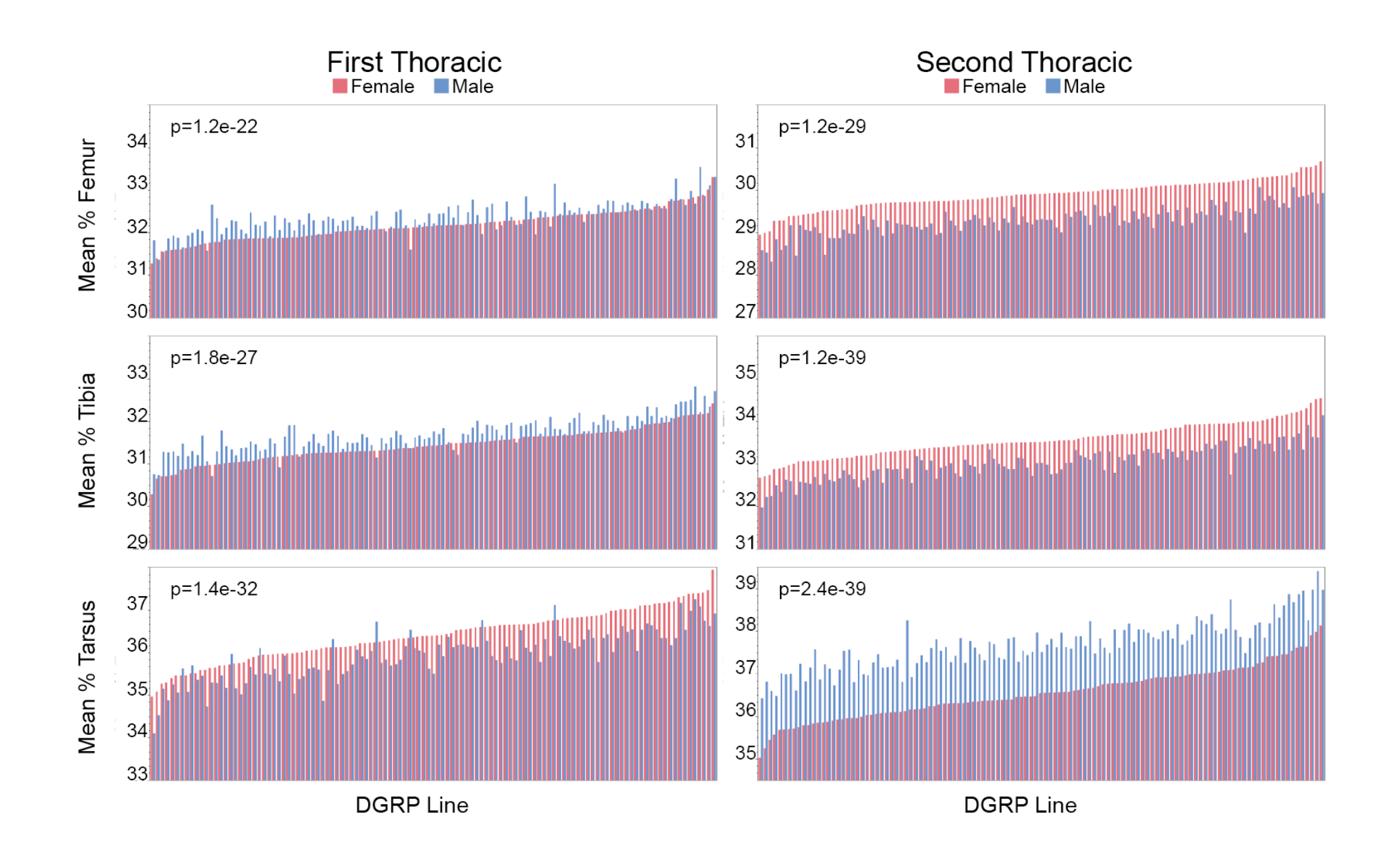

Supplement: Figure S2 — Variation in proportions of leg segments. Mean leg segment proportion for each measured DGRP line is graphed on the Y-axis, organized from smallest to largest based on female values (red) for each trait. Males are shown in blue. The P-value for line effect is also shown, and was very significant in all cases. (TIF) [file pone.0060261.s002.tif]
